# The Human Species

**Published:** 1909-12

**Authors:** 


### Reviews of Books.

**The Human Species.** By LUDWIG HOPF. (Authorised English Edition.) Pp. xx, 457. London: Longmans, Green & Co. 1909.

The author's aim in this most comprehensive book, as stated in the preface, is " the comparison of the essential characteristics of man with those of lower animals in the light of the results of recent research."

The author commences with a historical account of the opinions held in various countries and at various periods concerning the origin of man, and then goes on to discuss the present distribution of mankind, and whether man is to be regarded as constituting one or more than one species. He then considers the question as to the ancestors of man, and whether we have proof of his existence in the Tertiary period, concluding that we have as yet no proof of this. Then follow 130 well-illustrated pages devoted to comparative anatomy and histology, and these are succeeded by about 70 pages dealing with comparative physiology. A most interesting and comprehensive psychological section succeeds with reference to such subjects as emotions and their expression, social conditions and observances, religion, arts and handicrafts, painting, music, writing.

The last section of the book is concerned with comparative pathology and pathological anatomy, and after a somewhat brief account of abnormalities and monstrosities a long series of infectious and non-infectious diseases is discussed, and in the great majority of cases it appears that man shares the liability to attack with the lower animals.

The English translation seems to be very well carried out, the illustrations are good and numerous, and the printing and general get up of the book are all that could be desired.

